# The development of a low-cost photogrammetry-based 3D hand scanner

**DOI:** 10.1016/j.ohx.2021.e00212

**Published:** 2021-06-21

**Authors:** Yusheng Yang, Jun Xu, Willemijn S. Elkhuizen, Yu Song

**Affiliations:** a Delft University of Technology, The Netherlands; b Shanghai University, China

**Keywords:** 3D scan, Photogrammetry, Raspberry Pi

## Abstract

Acquiring an accurate 3D scan of the human hand is a challenging task, mainly due to the complicated geometry and the instability of the hand. In this paper, we present a low-cost photogrammetry-based scanner that is designed for scanning the human hand. The scanner has fifty modules, each has a Raspberry Pi with an 8-megapixels camera. They are uniformly positioned in two parallel frames and 96% of a hand surface can be viewed by at least 3 cameras. Using the timestamp method, we synchronize the shutters of the 50 cameras within the range of 80 ms to minimize the influence of the instability of the hand. Moreover, the scanner is easy to build with its modular design, and easy to operate with a laptop that is connected to the system by WiFi. Using a 3D printed prosthetic hand, we compared the 3D scanning accuracy of the proposed scanner with the Artec Spider® scanner. The mean absolute error between the two scans is 0.62 ± 0.28 mm. It is concluded that the proposed hand scanner can be used as a low-cost yet accurate tool in many applications, such as personalized product design.

## Graphical abstract

**Figure:**
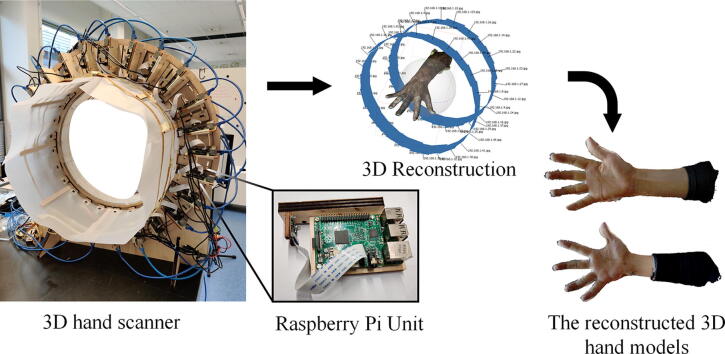

**Specifications table:** 

Hardware name	3D human hand scanner
Subject area	1. Engineering and Material Science 2. Educational Tools and Open-Source Alternatives to Existing Infrastructure
Hardware type	1. Imaging tools 2. Electrical engineering and computer science 3. Mechatronics
Open Source License	Creative common 4.0
Cost of Hardware	About 3650 Euros
Source File Repository	https://doi.org/10.17632/7rcpk2trr7.1

## Hardware in context

In the area of human integrated digital twin [1], 3D scanning of the human body plays an important role in bridging the physical and the digital worlds. Among different parts of the human body, the hand, which consists of 27 bones and has 27 degrees of freedom, is one of the most complex structures [2]. While it is a marvel of dexterity, it also posts challenges on the digitization techniques, especially for the low-cost 3D scanning system. For instance, the hand is not always stable [3], therefore the digitization speed should be as fast as possible [4].

In the past decades, many technologies have been developed and used in digitizing the 3D shape of (part of) the human body, e.g. structured light, time-of-flight scanning, laser scanning, computed tomography and photogrammetry. In the area of high-speed digitization of human body shapes, a typical example is the 3dMD® scanning system [5], which is built on multiple Modular Camera Units (MCUs) utilizing a hybrid of stereo photogrammetry and structured light technology. The system can be configured to capture the shape of the whole body as well as different parts, depending on the needs of the applications. Though the system can reach an accuracy of 0.7 mm at a scanning speed up to 120 frames per second (FPS) [5], the cost is high (>€150,000 for a full-body scanner).

Technology advancements in electronics and embedded systems introduce new possibilities for 3D data collection, such as using Intel RealSense®, Azure Kinect®. The Intel RealSense® is a low-cost (~200 euros) imaging device based on structured light and stereo matching technologies. It can capture color and depth images simultaneously in high FPS (up to 90 FPS), and the depth accuracy can reach 1.48 ± 0.28 mm and 1.46 ± 0.26 mm regarding scanning the facial palsy and the healthy face, respectively [6]. The Azure Kinect (~400 euros) was recently released by Microsoft® for computer vision applications [7]. It utilizes the time-of-flight technique and is able to acquire one-megapixel depth images at a speed up to 30 FPS. However, both Intel Real Sense® and Azure Kinect® can only capture the 3D information of the object from one side, it requires several of them to reconstruct the whole shape of the object, and the cost and the complexity of the system increase accordingly.

Among different techniques, close-range photogrammetry has the advantages such as simple requirements (only images are required), short data acquisition time, high accuracy, and its noninvasive nature, which makes it an appropriate approach for scanning (part of) the human body. For instance, in the Botscan system developed by Botspot for scanning the human body, 64 Canon EOS 1200Ds cameras (~500 euros for each camera) are utilized where 60 of them are evenly distributed around the center of the scanning area and the remaining four are mounted at the top [8]. However, the cost of the system is also high, and the system needs to be re-configured for scanning different body parts/objects, which introduces an extra level of complexity.

A fast, convenient and low-cost 3D hand scanner can promote the computational design of hand-related personalized products, such as gloves [9] and handles [10], facilitate ergonomics studies [11], accelerate the development of different Human-Computer Interaction (HCI) tools for virtual/augmented reality applications, etc. However, few studies to date have focused on building a low-cost scanner for the human hand.

In this paper, we address this gap by developing a photogrammetry-based scanning system to capture the 3D shape of the human hand. We set the requirements of the system as: 1) it should be able to capture the full shape of the human hand; 2) its design should be modular and it can be easily and quickly assembled; 3) it should be built at an affordable price; 4) it should be able to complete the scan in a very short time span (e.g. 100 ms) to avoid the influence of the shaking of the hand; 5) it should have a high accuracy (less than 1 mm) and 6) it should be easy to operate by the user.

## Hardware description

**Fig. 1:**
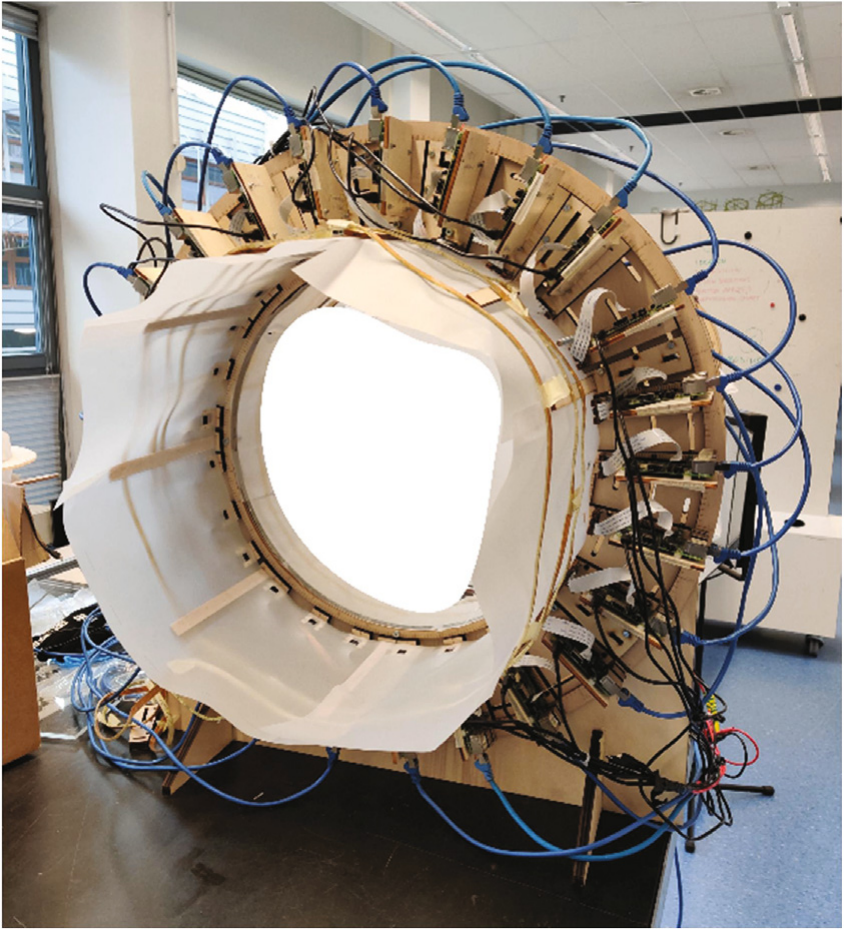
An overview of the hand scanner.

The proposed hand scanning system (Fig. 1) consists of three major parts: the electronics part, the mechanical structure, and the software part. Except the power supply and the data processing module, the main components of the electronic part are mounted on the cylindrical mechanical structure, which by its modularity can easily be assembled and adjusted. The software part is also referred to as the “control system”, which can receive commands from the operator and handle them accordingly.

### Electronics parts

**Fig. 2:**
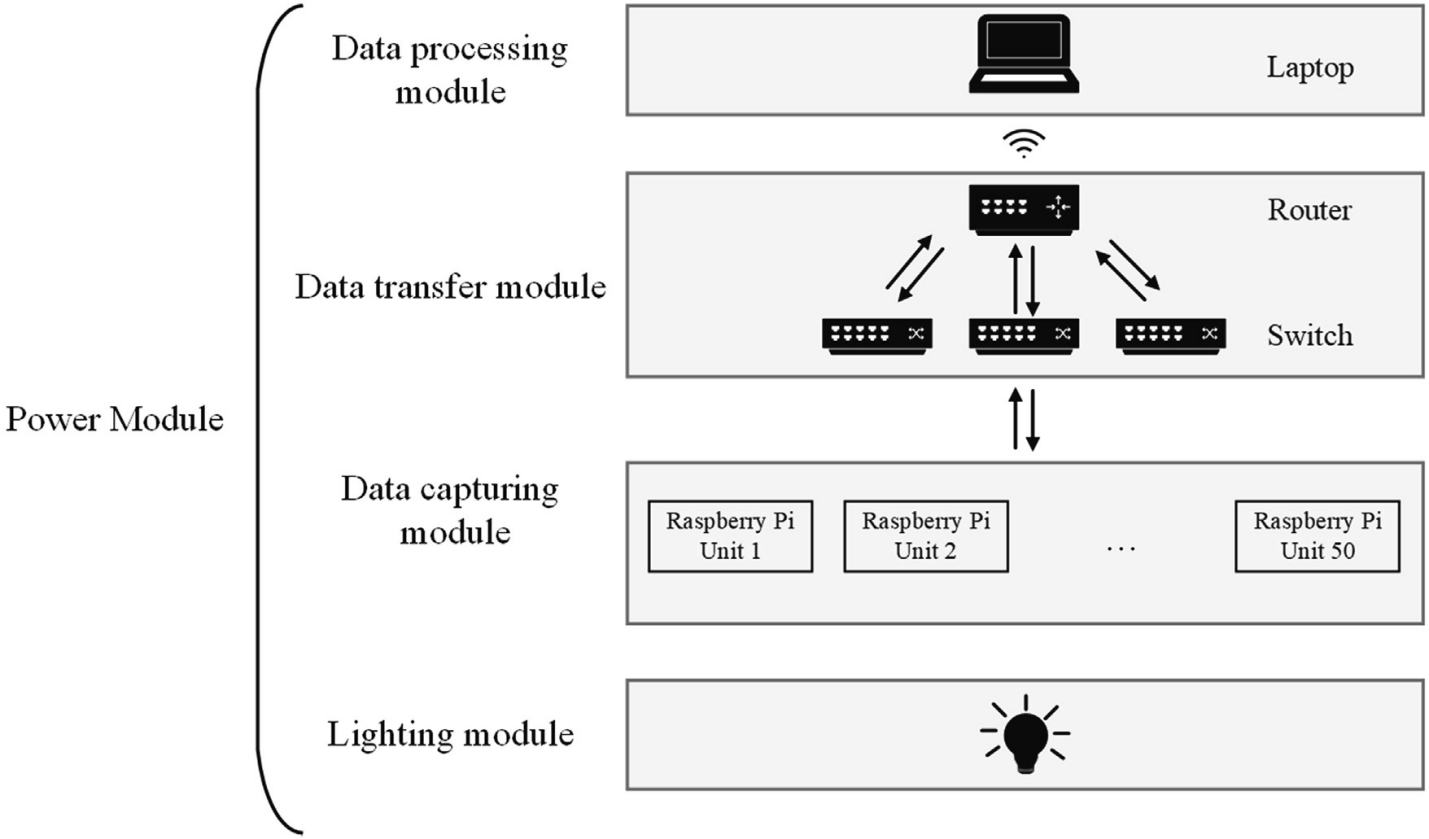
An overview of the electronic design for the hand scanner.

The electronics part of the hand scanner consists of five modules (Fig. 2): 1) the data capturing module; 2) the data transfer module; 3) the data processing module; 4) the lighting module and 5) the power module. The data capturing module has fifty Raspberry Pi units for capturing images of the hand from different directions. Each Raspberry Pi unit is made of a Raspberry Pi 2B V1.1 single-board computer (incl. an 8 GB microSD card) and a Raspberry Pi camera module V2.1, where they are connected through the FPC connector on the Raspberry Pi board [12]. The horizontal and vertical angles of view for the camera module are 62.2 and 48.8 degrees, respectively. The focal length of the camera is 3.04 mm and the camera is able to capture an image at a resolution of 3280×2464 pixels.

**Fig. 3:**
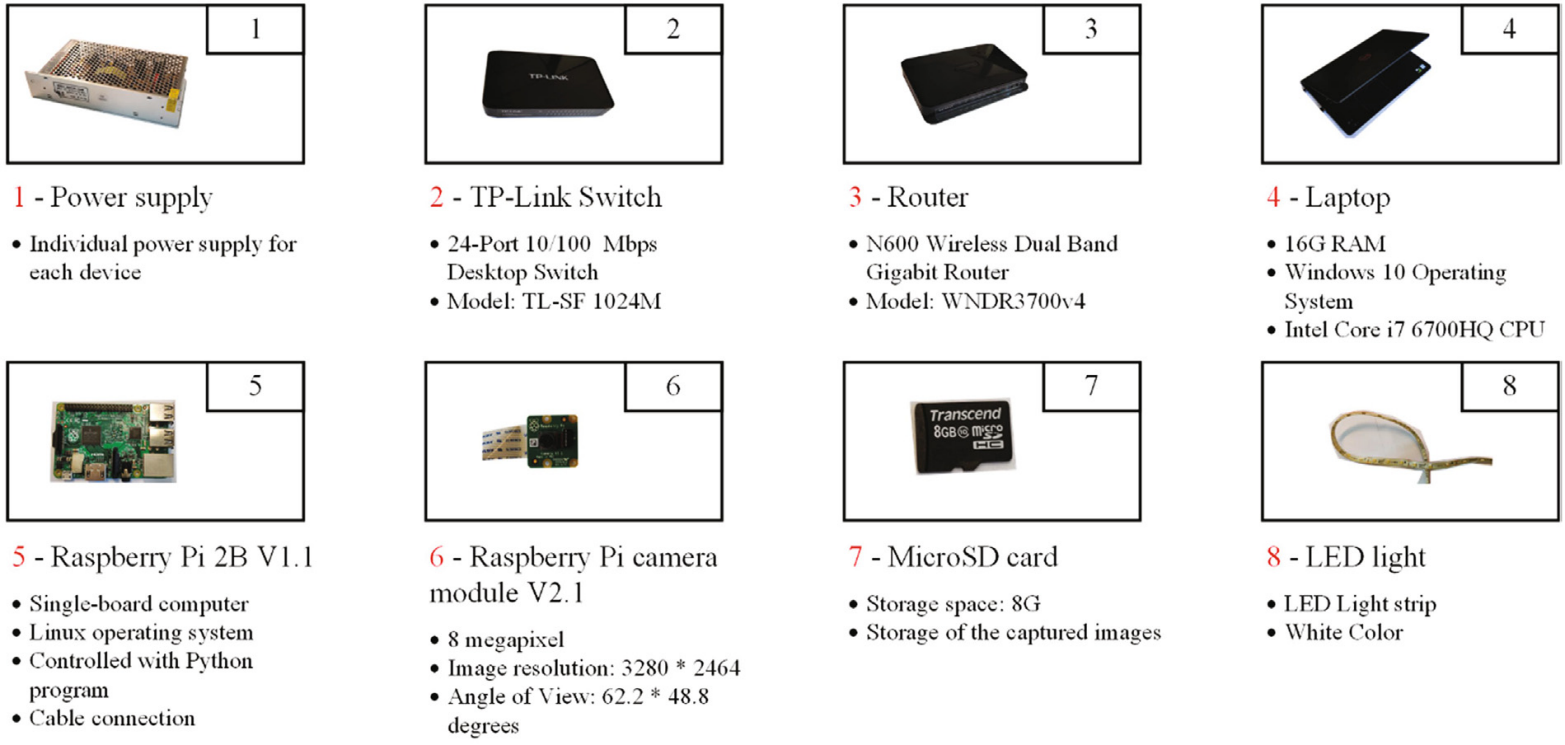
Electronic components.

The data transmission module has three 10/100 Mbps TP-Link® desktop switches and one N600 wireless dual band gigabit router (Brand: NETGEAR®). The Raspberry Pis are linked to the switches through the Ethernet ports. The wireless router links all switches and also acts as the interface to the data processing module. A Dell® Precision 7559 laptop with Intel® Core i7® CPU is used as the controller as well as the device for data post-processing. However, the laptop can be replaced by any computer which is able to conduct 3D reconstruction based on photogrammetry. In order to increase the shutter speed for a fast image acquisition process, three white LED strips are winded around the scanners as the light source. Together with the mechanical structure, a soft box is formed for more uniform illumination. The power supply units supply different voltages for different devices according to the requirement of each device. Specifically, the Raspberry Pi units are powered by 5 V, as for the switch and the router, 9 V and 12 V are needed, respectively. Fig. 3 gives an overview of all electronic components.

### Mechanical design

**Fig. 4:**
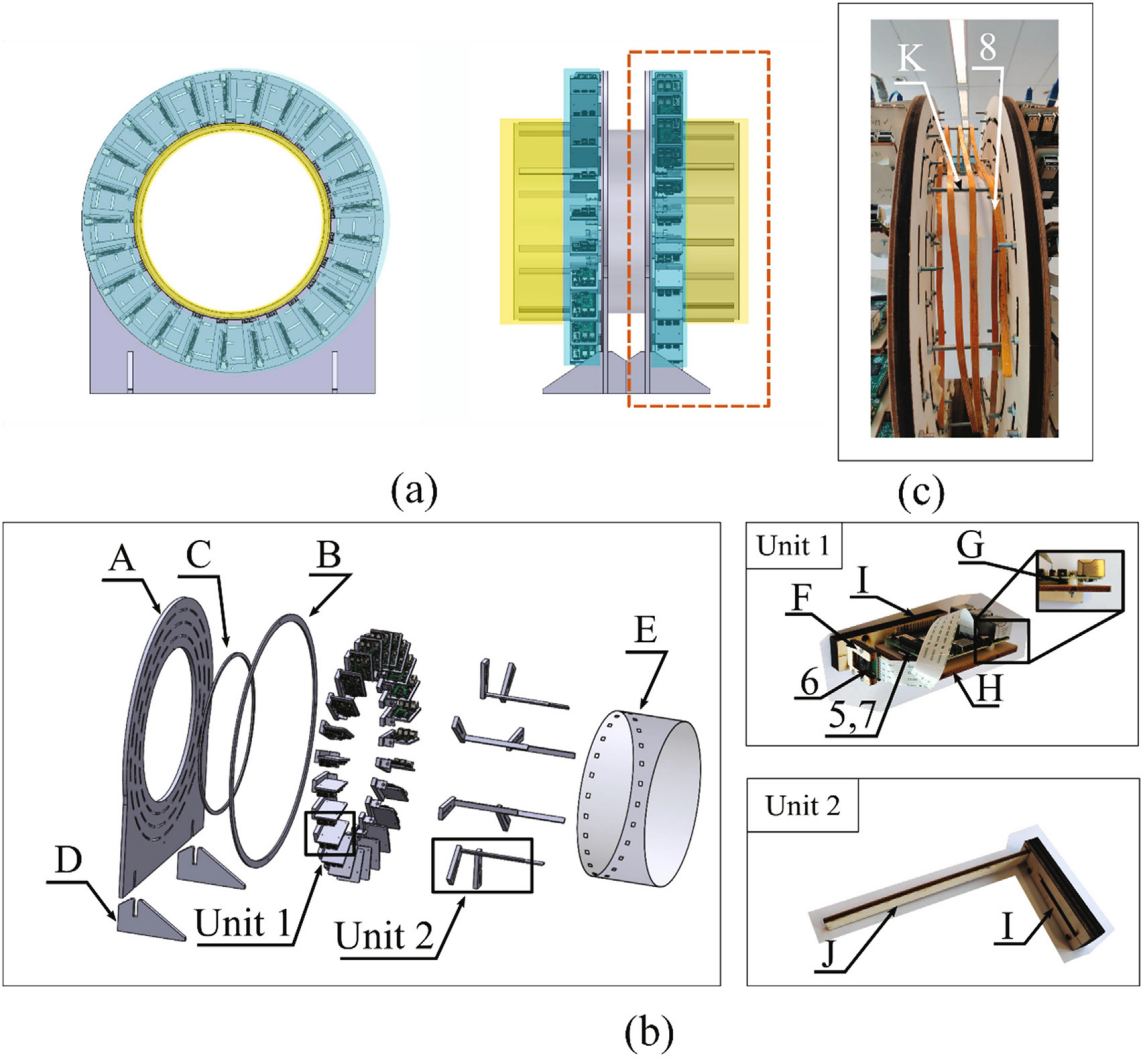
An overview of the mechanical design for the hand scanner: (a) The mechanical structures to support the data capturing module (blue) and the lighting module (yellow). The rectangle part represents one of the two identical frames. (b) All components for one frame. Each component is labeled with a letter except the electronics components, which are indexed by the same numbers in Section 2.1. (c) The threaded rods between two frames. (For interpretation of the references to color in this figure legend, the reader is referred to the web version of this article.)

As illustrated in Fig. 1, the hand scanner is placed on a table with electronic components such as the power supply module, the switches and the router. Since the data processing module, i.e. the laptop, can access the hand scanner through WiFi, it can be placed anywhere in the range of the router. The mechanical design supports the data capturing module and the lighting module, as shown in Fig. 4(a). The fifty Raspberry Pi units in the data capturing module are mounted inward, onto two parallel rounded frames. The two frames each holding 25 Raspberry Pi units are mirrored and connected by six threaded rods (K) with a distance of 83 mm as Fig. 4(c). The dimensions of the frame and the distance between two frames are optimized so that 96% of the hand surface can be viewed by at least 3 cameras, providing that the hand is positioned approximately in the middle of the scanner with a “standard posture”, where the palm is kept flat with wide spread fingers to ensure the visibility of the finger crotches. More details about the calculation of the dimensions can be found in [13].

All mechanical components in a single frame are labeled by letters in Fig. 4(b), and the electronic components are labeled by their indexes as described in Section 2.1. A frame plate (A) is supported by two frame stands (D) and holds 25 Raspberry Pi units (Unit 1) and eight lighting holders (Unit 2). In a Raspberry Pi unit (Unit 1), a Raspberry Pi (5) is fixed on an “L” shape wooden board (H), and four plastic washers (G) are installed between them to prevent direct contact with the PCB. A “U” shape wooden board (F) serves as the holder of the Raspberry Pi camera module V2.1 (6). Both the “L” shape and the “U” shape wooden boards (H, F) are fixed on the sliding wooden board (I). The position of the sliding board (I) is restricted by two ring constraints (B, C) and the position can be easily adjusted along the frame plate (A) by sliding the screws along the slots on the frame plate (A). For each ring constraint, 360 evenly distributed landmarks are engraved on the ring surface for positioning the sliding board (I) at the right angle. With this structure, the positions of all cameras can be adjusted easily for ensuring the lens of cameras are positioned toward the center of the ring constraint. For the lighting holders (Unit 2), the same sliding board (I) is used to hold the wooden beam (J), which is used to support the white translucent paper (E). Together with the LED lights, they form a soft box for the camera to acquire better images.

### Software system

The software system of the hand scanner includes two parts, the client on the Raspberry Pi unit and the command part on the laptop, both coded with Python. When the scanner is powered on, each Raspberry Pi unit is assigned with a unique IP address by the router, thus each unit can be controlled individually. The client on the Raspberry Pi units starts automatically and listens to the commands from the laptop. Before data acquisition, all Raspberry Pi units need to synchronize their clock with the laptop through the network time protocol (NTP). For capturing images simultaneously, a timestamp, which is three seconds later than the time that the command is sent, is coded in the “capture image command” sent by the laptop. The “three seconds later” is designed to tolerate delays of the network. Once the client received the command of capturing the image, it will capture the image at the time specified by the timestamp with a resolution of 3280 × 2464 at ISO 100. In this way, it is guaranteed that all Raspberry Pi modules will capture the images of the hand at the same time (in our case it was measured within 80 ms range). The laptop can also send other commands such as uploading images, shutting down, rebooting, to each Raspberry Pi.

## Design files

### Design files summary

**Table:** 

Design file name	File type	Open source license	Location of the file
Frame plate.DXF	Laser cutter file	Creative common 4.0	*available with the article*
Ring constraint 1.DXF	Laser cutter file	Creative common 4.0	*available with the article*
Ring constraint 2.DXF	Laser cutter file	Creative common 4.0	*available with the article*
Frame stand board.DXF	Laser cutter file	Creative common 4.0	*available with the article*
Sliding wood board.DXF	Laser cutter file	Creative common 4.0	*available with the article*
“L” shape wood board.DXF	Laser cutter file	Creative common 4.0	*available with the article*
“U” shape wood board.DXF	Laser cutter file	Creative common 4.0	*available with the article*
Wood beam.DXF	Laser cutter file	Creative common 4.0	*available with the article*
Final assembly.zip	Zip of all CAD files	Creative common 4.0	*available with the article*
Hand scanner scripts.zip	Zip of Python scripts	Creative common 4.0	*available with the article*

1. Frame plate.DXF: The file used for laser cutting the shape of the frame plate (A). The thickness of the wooden board is 12 mm.
2. Ring constraint 1.DXF: The file used for laser cutting the shape of the ring constraint 1 (B). The thickness of the board is 5 mm.
3. Ring constraint 2.DXF: The file used for laser cutting the shape of the ring constraint 2 (C). The thickness of the board is 5 mm.
4. Frame stand board.DXF: The file used for laser cutting the shape of the frame stand board (D). The thickness of the board is 12 mm.
5. Sliding wood board.DXF: The file used for laser cutting the shape of the sliding wooden board (I). The thickness of the board is 12 mm.
6. “L” shape wood board.DXF: The file used for laser cutting the shape of the “L” shape wooden board (H). The thickness of the board is 5 mm.
7. “U” shape wood board.DXF: The file used for laser cutting the shape of the “U” shape wooden board (F). The thickness of the board is 5 mm.
8. Wood beam.DXF: The file used for laser cutting the shape of the wooden beam (J). The thickness of the board is 5 mm.
9. Final assembly.zip: The zip file contains the SolidWorks® files of the design.
10. Hand scanner scripts: The zip file contains all the code necessary to operate the hand scanner.

## Bill of materials

### Bill of materials

**Table:** Table Bill of Materials

Designator	Component	Number	Cost per unit -currency	Total cost -currency	Source of materials	Material type
P1	Raspberry pi 2B V1.1	50	37.95€	1,897.5	KIWI electronics	Other
P2	Raspberry pi camera module V2.1	50	19.15€	957.5	Amazon	Other
P3	Plastic washer	200	0.19€	38	BoltWorld	Other
P4	Translucent Vellum Paper	1	23.98€	23.98	Stationery shop	Other
P5	LED light strip, white color	3	27.99€	83.97	Amazon	Other
P6	Threaded rods M5 * 250 mm	6	7.47€	44.82	Amazon	Other
P7	Pan head screws M5 *50	148	0.11€	16.28	screwFix	Other
P8	Screw nuts M5	148	0.04€	5.92	screwFix	Other
P9	Round head cap screw bolts M2*16	400	0.03€	12	Amazon	Other
P10	Screw nuts M2	400	0.03€	12	AliExpress	Other
P11	Micro-SDHC 8 Gb	50	3.49€	174.5	Amazon	Other
P12	Multiplex Birch 6 mm wood board	2 m^2^	5.90€	11.8	Houthandel Online	Other
P13	Multiplex Birch 12 mm wood board	2 m^2^	9.68€	19.36	Houthandel Online	Other
P14	Double-sided tape	1	5.85€	5.85	Amazon	Other
P15	Network cable * 2 m	50	1.65€	82.5	Cable shop	Other
P16	Network cable * 0.5 m	3	1.09€	3.27	Cable shop	Other
P17	5 V power supply	4	34.99€	139.96	Amazon	Other
P18	Router	1	43.21€	43.21	AliExpress	Other
P19	Switch	3	27.13€	81.39	Amazon	Other

## Build instructions

### Construction of components

The ring constraints, “L” and “U” shape wooden boards and wooden beams are made of 5 mm multiplex wooden board. All other wooden components are made of 12 mm multiplex wooden board. A laser cutter (brand: Lion lasers) is used to manufacture those components. Benefited from the modular design principles, all Raspberry Pi units (Unit 1) and lighting holders (Unit 2) can be constructed independently before assembling them to the scanner. In the following section, the steps for assembling an individual Raspberry Pi unit (Unit 1) and the lighting holder (Unit 2) will be illustrated, then the procedure of assembling the scanner is presented.

### Assembling the Raspberry Pi unit

1. Install the SD card (P11) to the Raspberry Pi (P1).
2. Fix the Raspberry Pi (P1) on the “L” shape wooden board (H) with four M2 screws (P9) and nuts (P10), where four plastic washers (P3) are used (Fig. 5) to separate the PCB and the board. Once finished, this part (Part 1) is ready to be integrated into the Raspberry Pi unit;

**Fig. 5:**
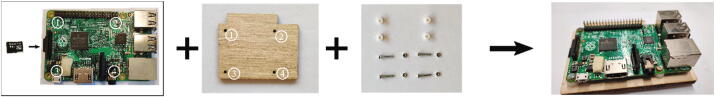
Assembling Part 1 of the Raspberry Pi holder.
3. Fix the Raspberry Pi camera module (P2) on the “U” shape wooden board (F) with M2 screws (P11) and nuts (P12) as shown in Fig. 6. This is Part 2 of the Raspberry Pi unit.

**Fig. 6:**
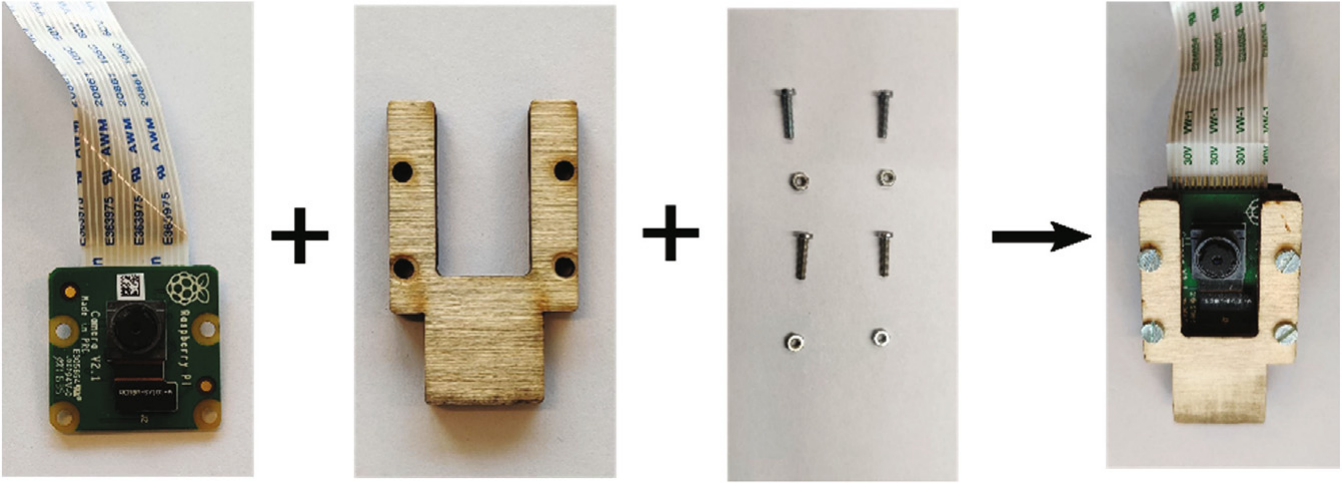
Assembling the Raspberry Pi camera module.
4. Installing Part1 and Part2 of the Raspberry Pi unit on the sliding wooden board (I) as shown in Fig. 7.

**Fig. 7:**
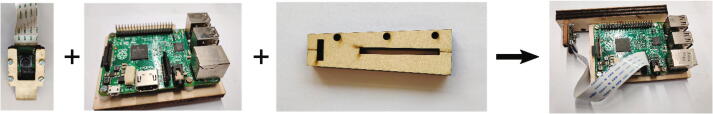
Assemble the Part 1 and Part 2 together as the Raspberry PI unit.

### Assembling the lighting holder

**Fig. 8:**
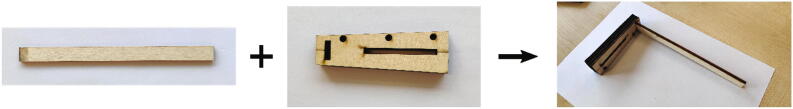
Assembling the lighting holder.

The base of the lighting holder is made of the same sliding wooden board (I) as the Raspberry Pi holder. It is assembled with the wooden beam (J) similar to the Raspberry Pi camera module as Fig. 8.

### Assembling the main body of the hand scanner

Since the hand scanner contains two identical frames, we only introduce the construction procedure of one individual frame, the other frame can be constructed with the same procedure. The procedure for assembling a frame includes the following steps:

1. Connect the frame stands (D) and the frame plate (A) as Fig. 9(a).

**Fig. 9:**
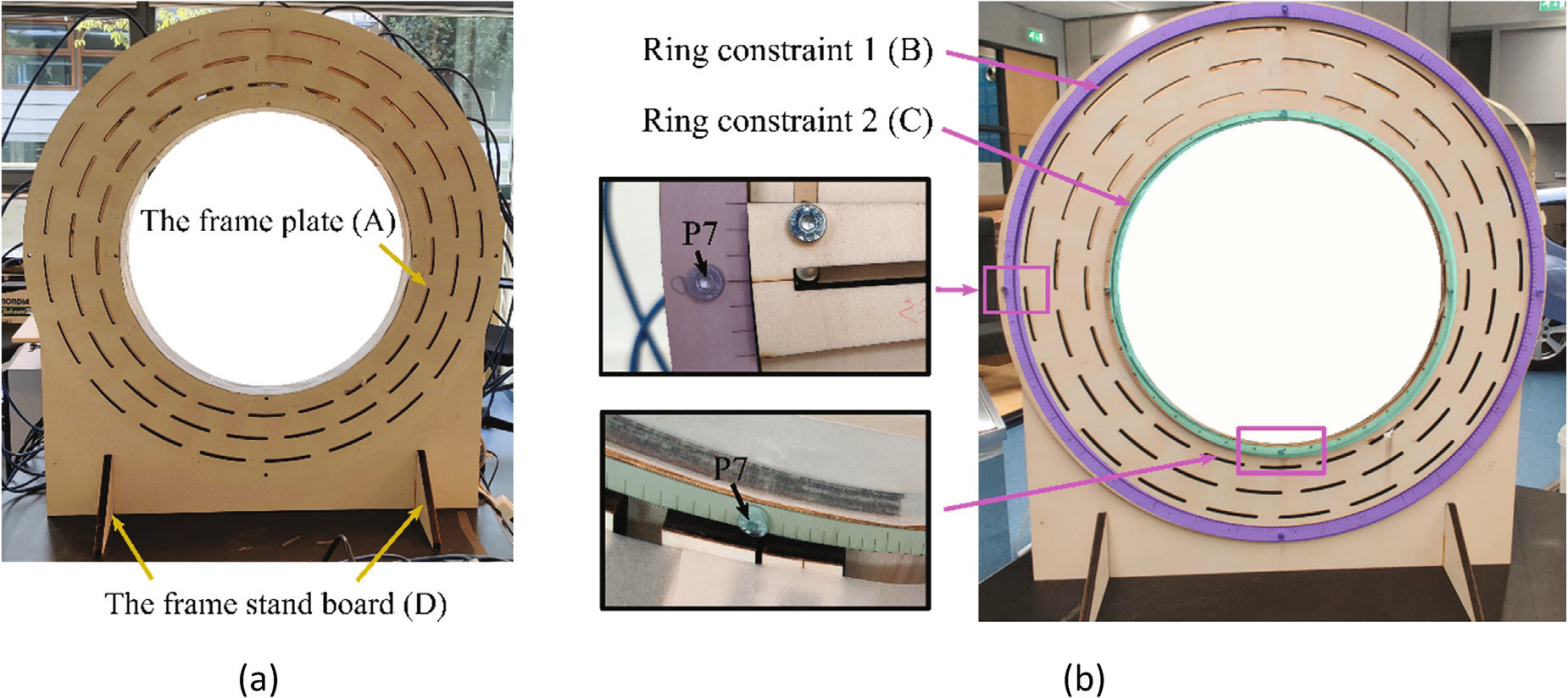
Assembling the frame plate and the constraint rings.
2. Fix two ring constraints (B, C) on the frame plate (A) with M5 screw bolts (P7) and nuts (P8) as shown in Fig. 9(b).
3. Install the Raspberry Pi unit (Unit 1) on the frame plate (A) as shown in Fig. 10(a). There are three staggered slots on the frame plate (A) along three concentric circles. It was designed that for any lines in the radial direction, it will cross at least two of the three staggered slots. The installation position of Unit 1 is defined by aligning the landmarks on the sliding wooden board (I) with the landmarks on the two ring constraints (B, C) to guarantee that the camera module (6) faces towards the center of the scanner as illustrated in Fig. 10(b). In this setup, at least two slots are available for the screw (M5 (P7)) to pass through the sliding wooden board (I) and the frame plate (A) for fixation. During installation, the angle between two adjacent Raspberry PI units is approximately 14.4 degrees to ensure that the 25 Raspberry Pi units are evenly distributed around the frame as Fig. 10(a).

**Fig. 10:**
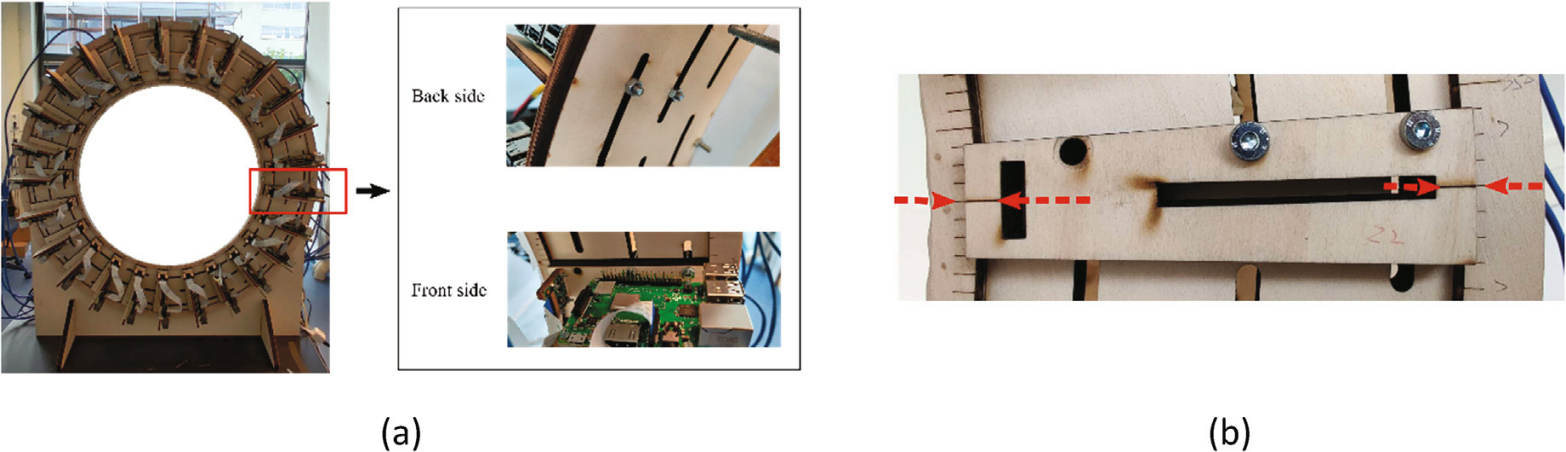
Assembling the Raspberry Pi units on a frame.
4. Fix eight lighting holders (Unit 2) on the frame plate (A). The lighting holders (Unit 2) are usually positioned in the middle of two adjacent Raspberry Pi units (Unit 1). In some cases, the installation position of the lighting holder might conflict with the Raspberry Pi unit (Unit 1). As the main purpose of the lighting unit is to support the translucent vellum paper to form a soft box and the needed accuracy of installation is not high, we install the lighting holders at the nearest available place. Since the lighting holders (Unit 2) use the same sliding wooden board (I) as the Raspberry Pi holders (Unit 1), the same M5 screws (P7) and nuts (P8) are used for fixing it on the frame as Fig. 11.

**Fig. 11:**
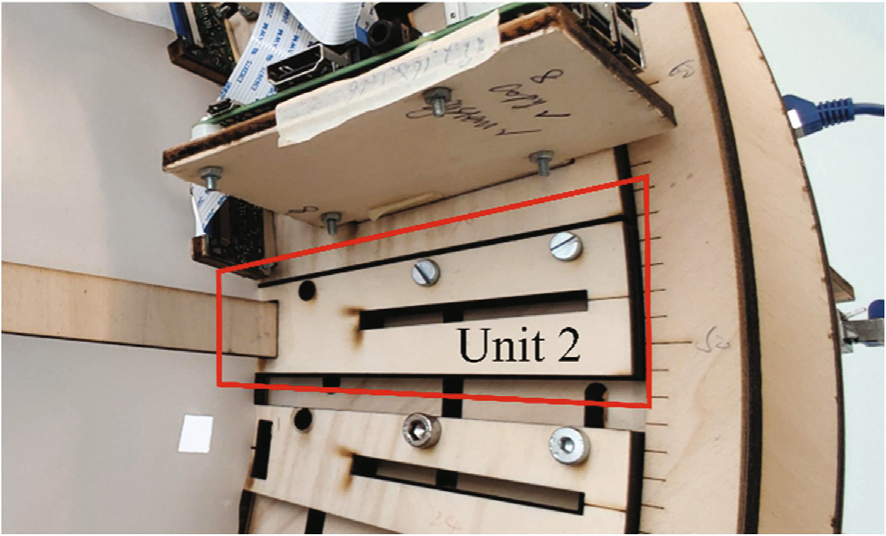
Installing the lighting holder to the frame.

### Assembling two frame plates together

**Fig. 12:**
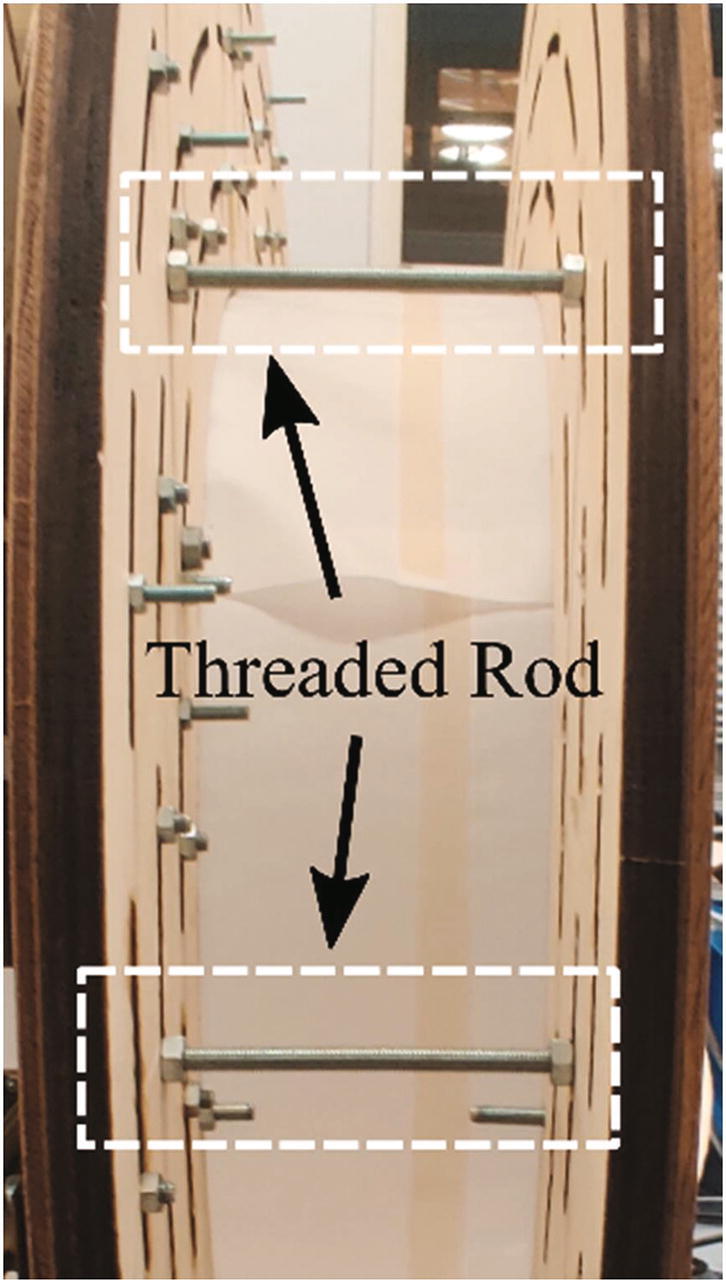
Assembling two frames together.

Following the above steps, two frame plates (A) can be assembled. Next, they are placed back-to-back, and six M5 threaded rods (P6) are used to fix the relative position (distance is 83 mm) of frame plates as shown in Fig. 12.

### Glue the translucent vellum paper

**Fig. 13:**
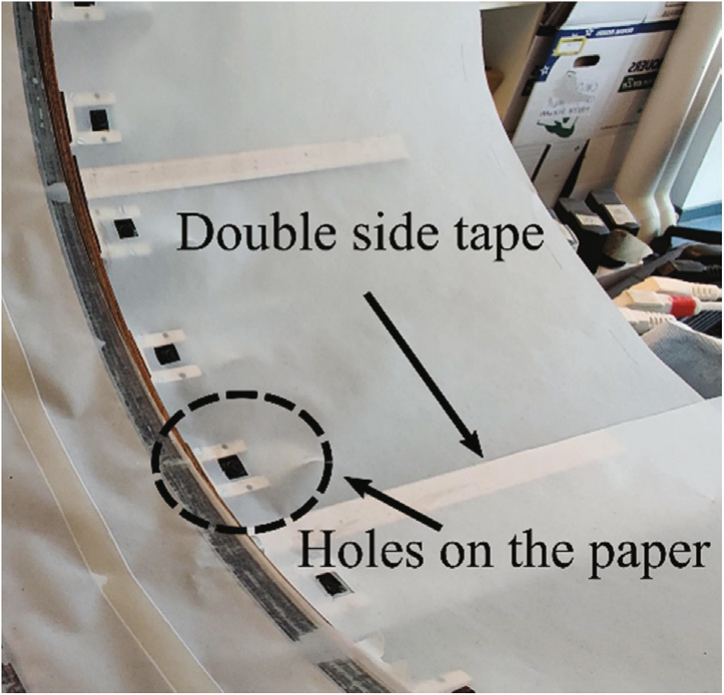
Assembling the lighting bearers.

To install the light diffuser, we stick the translucent vellum paper on the lighting holder (Unit 2) with double side tapes (P14). To avoid blocking the view of the lens of cameras, we cut holes on the paper (P4). The size of the holes is 10 mm×10 mm. To form the effect of a complete soft box, areas between two frames are covered with the translucent vellum paper (P4) as well (Fig. 13).

### Wind the LED strip around the hand scanner

**Fig. 14:**
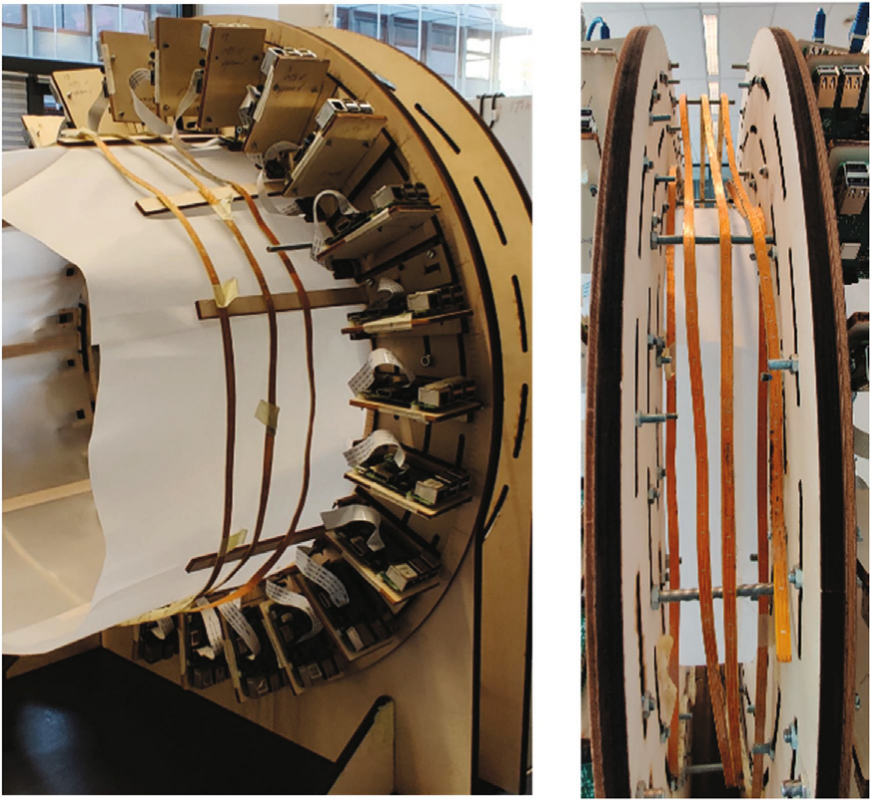
Winding the LED strip.

Three light strips (P5) are winded around the two light holders (J) and the area between two frames, respectively. In the area between two frame plates, the threaded rods (K) are used to support the LED strip as Fig. 14.

### Connecting the cables

In this step, each Raspberry Pi (P15) is connected to the switch with Ethernet cables. The power for Raspberry Pi units is also supplied with micro USB cables. As in the design, the Ethernet ports of all Raspberry Pis can be easily accessed, it is convenient to connect the cables and manage the cables with tie wraps.

## Operation instructions

### Configure each Raspberry Pi

Each Raspberry Pi should be configured after assembling it to the Raspberry Pi unit (Section 5.2).

1. Each Raspberry Pi is operated by the Raspbian Operation System (OS) which is installed on the SD card. To install the Raspbian OS on the SD card, we followed the instruction in [14];
2. After the OS is installed on the SD card, we insert the card into the Raspberry Pi;
3. Start the Raspberry Pi, and create a new folder named “scanner” in the home folder (the default path is “/home/pi/”). Download the programs about the scanner (“Hand scanner scripts.zip”) and copy the files inside the folder “pi_code” to the newly created “scanner” folder;
4. Set up the autostart of the program:
  a) Open a terminal and type the command “sudo nano/etc/rc.local”, this will open the “rc.local” file with the editor named “nano”;
  b) Add the following commands in “rc.local” file, “sudo python3/home/pi/scanner/scanner_pi.py &”. Where “/home/pi/scanner/scanner_pi.py” is the path to the program “scanner_pi.py” and the ampersand “&” means that the program runs in a separate progress. The commands should be inserted before the line “exit 0”;
  c) Exit the editor by pressing CTRL + X and Y.
5. Restart the Raspberry Pi.
6. Repeat the procedure 1–5 for all 50 Raspberry Pis.

The program on the Raspberry Pi starts automatically when the hand scanner is powered ON (i.e. rebooted) and runs as a slave waiting for the commands from the controller (the laptop).

### Configure the laptop and network

1. The Python3 environment is required for the laptop to run the program. To install Python3, the user can follow the steps described in [15].
2. Create a new folder and download the code for the scanner (“Hand scanner scripts.zip”) to the laptop, copy the files inside the folder “pc_code” to the newly created folder;
3. Connect the laptop to the router wirelessly. The default name of the WiFi created by the router is “NETGEAR 2.4 G” and the default username and the password are “admin” and “password”, respectively;
4. Launch a browser and enter “http://www.routerlogin.net^”^ in the web address bar. Enter the home screen of the router with the same username and password of the WiFi;
5. Clicking the “Attached Devices” button on the home screen. The Attached Device screen shows all Raspberry Pis that connect to the router and their corresponding IP addresses;
6. Based on the information collected from the above steps, editing the configuration file “pcConfig.ini” which is also in the newly created folder. The configuration file contains the IP addresses of Raspberry Pis and it will be loaded first when the program is called (Section 6.3);

We suggested to switch on Raspberry Pi one by one in this process in order to link the IP address and the physical location of the Raspberry Pi module.

### Controlling the hand scanner from the computer

#### Check the network connection

Before sending commands to the hand scanner, we need to ensure that all Raspberry Pis are online. This can be done by opening a terminal on the computer, changing the current folder to the folder that contains the codes. Run the following program:

“Python .\autoPing.py”

the program will check the status of the connection between the computer and all Raspberry Pis. It will print the number of the connected Raspberry Pis and identify which one has a connection problem. In our practice, a connection problem is often caused by that the program in the Raspberry Pi does not start automatically. Restarting the corresponding Raspberry Pi might fix the problem. Otherwise, the procedure in Section 6.1 should be repeated on this particular Raspberry Pi module.

#### Acquire images of the human hand

The hand scanner can handle several commands, including capturing the image, shutting down, and rebooting. The commands are sent by running the same program with different input parameters. Before capturing the images, the subject to be scanned should stand in front of the hand scanner and place his/her hand at the center of the hand scanner. To acquire 50 images of the human hand, the operator needs to open a terminal on the laptop and run the following command:

“python .\scanner_pc.py cmd_capture”

The image capturing command will be sent to all Raspberry Pis through the network, and for each Raspberry Pi module, one picture will be captured three seconds later.

#### Fetch the acquired images

The images captured by the Raspberry Pis are stored locally and needed to be transferred to the computer for post-processing. The operator can fetch those images by typing the following command in the terminal:

“python .\loadFileFromPi.py capture”

The images on all Raspberry Pis will be downloaded to the same folder on the computer. The location of this folder can be specified in the program “loadFileFromPi.py”. The operator can also download the image from a specific Raspberry Pi by specifying the corresponding IP address as:

“pscp pi@{$ip_address}:/home/pi/scanner/image/capture.jpg {$folder_on_computer}”

Here {$ip_address} is the IP address of the specific Raspberry Pi, and {$folder_on_computer} is the location where the images are stored on the computer.

#### Other operations

If the operator wants to reboot the scanner, he/she needs to run the command “python .\ scanner_pc.py cmd_reboot”. Similarly, he/she can switch off the Raspberry PI by the command “.\ scanner_pc.py cmd_shutdown”.

### Reconstruct the 3D hand model

**Fig. 15:**
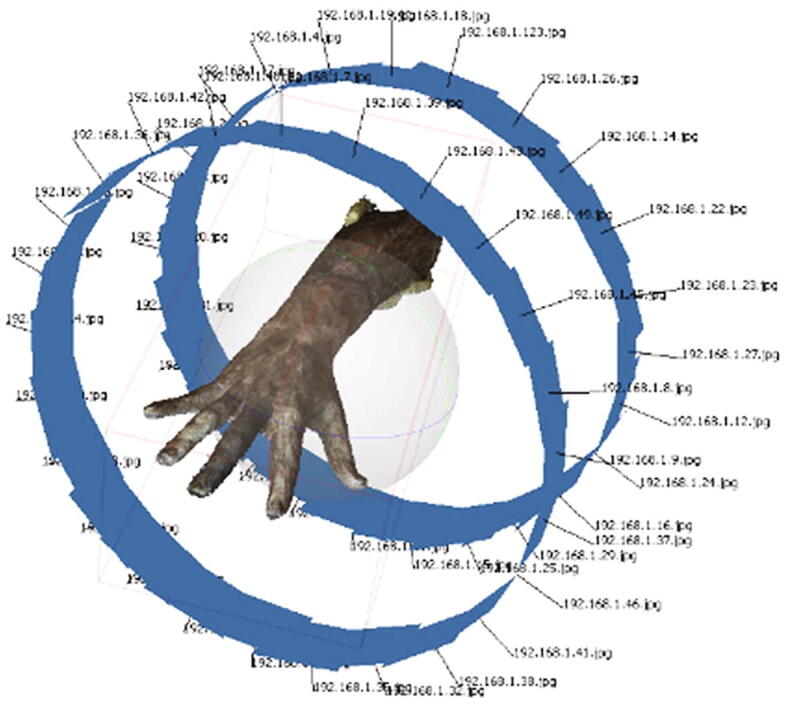
The 3D scan reconstructed using Agisoft® Photoscan®.

After uploading images from all Raspberry Pis, the 3D hand model can be reconstructed by software tools such as Agisoft® Photoscan® (64-bit version in this case). The workflow of reconstruction can be conducted in four steps [16]: 1) “Workflow -> Loading the image”; 2) “Workflow -> Aligning the photos”; 3) “Workflow -> Building dense cloud”. In this case, the user needs to change the parameter “Quality” to “Ultra high” for a high-quality result; and 4) “Workflow -> Build mesh”, where the user can change the parameter “Polygon count” to “High” for the best performance. A reconstruction result is shown in Fig. 15 where the 3D model and the camera positions are presented.

### Export the mesh

The mesh model reconstructed by the Agisoft® Photoscan® program can be exported in different formats, e.g. ply, obj or 3ds. A user can click “File->Export Model”, then choose the desired file format and the path, the 3D model will be saved on the computer and can be used for different purposes.

## Validation and characterization

### Accuracy of the scanner

**Fig. 16:**
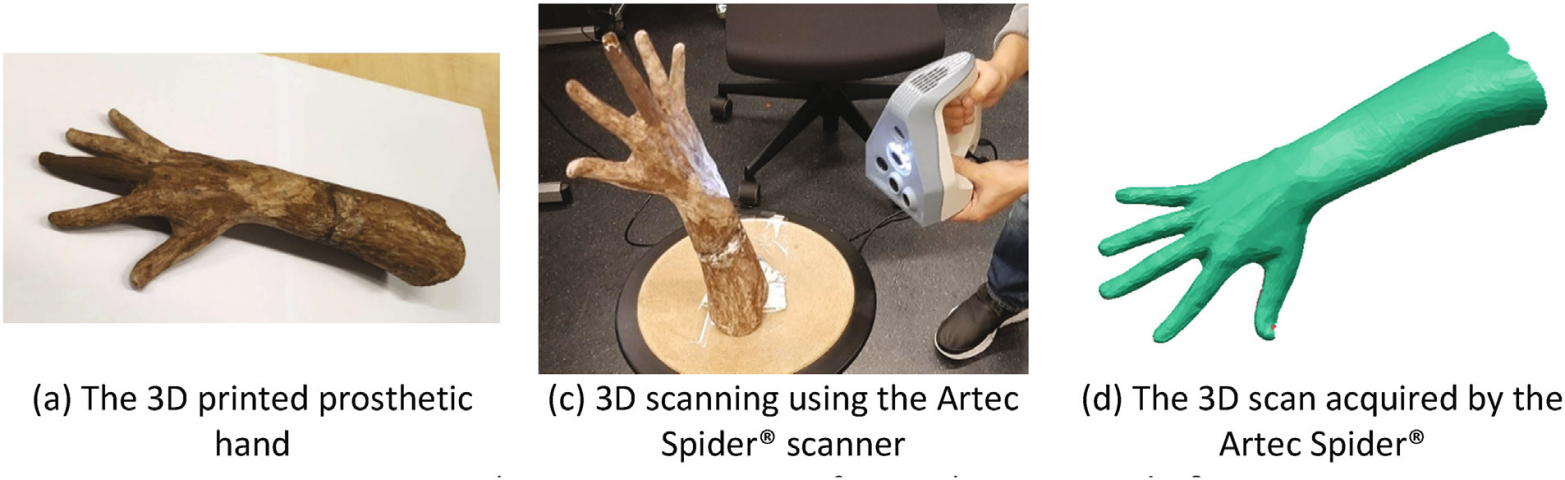
The scanning process of using the Artec Spider®.

The main function of the proposed hand scanner is to capture the images of the hand and reconstruct an accurate 3D hand scan based on those images. In order to verify the reconstruction accuracy of the designed hand scanner, we scanned a prosthetic hand using both the proposed scanner and a commercial scanner Artec Spider®. The reason for using the prosthetic hand is that we can actually compare the accuracy between scanners, as most commercial scanners only can capture a 3D scan of the hand from one view at a time, and for building a complete 3D hand model, many 3D scans from different view angles are needed. During this process, it is difficult for a subject to keep his/her hand in a fixed position, and even more difficult for being scanned by two scanners. As the prosthetic hand has a fixed shape, we selected the Artec Spider® scanner to build the reference model for its high accuracy (0.05 mm) [17]. Fig. 16(a) shows the 3D prosthetic hand which was printed by an Ultimaker® 3D printer and later painted to simulate the skin color. Using the Artec Spider® (Fig. 16(b)), we acquired the scan of the model as Fig. 16(c). The scanning process of using the Artec Spider® was approximately one hour (incl. post-processing), and the acquired scan is used as the “reference” model.

**Fig. 17:**
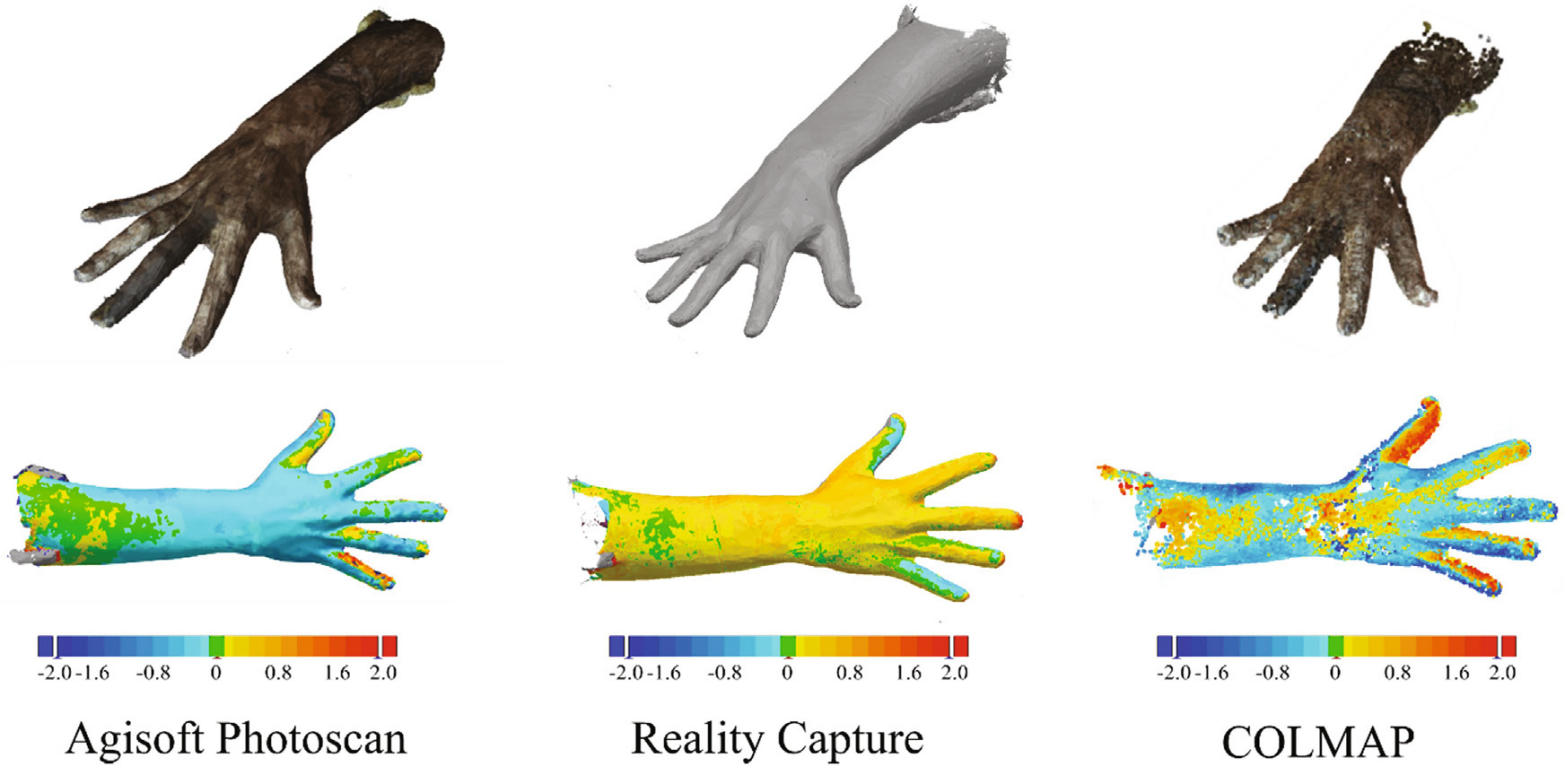
The 3D models reconstructed by different software tools (Top) and the comparison between the reconstructed model and the model captured by the Artec Spider® (Bottom).

**Fig. 18:**
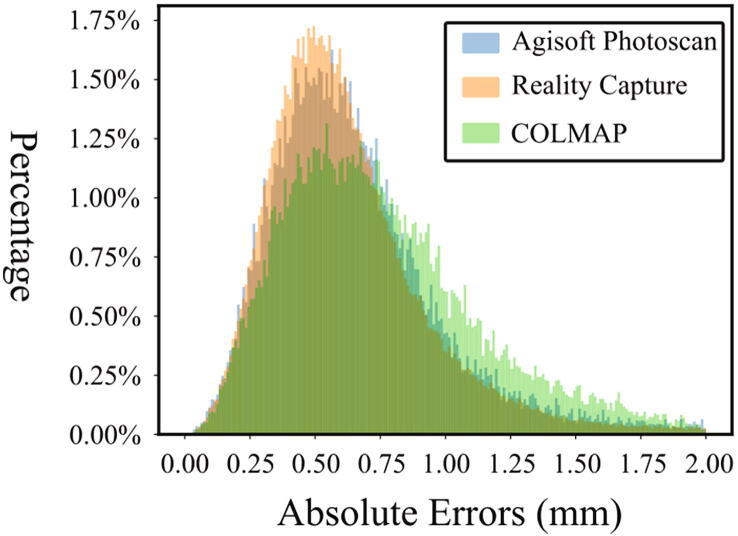
The histogram of absolute errors of the three models regarding the “reference” model.

In the process of building a 3D scan using photogrammetry, the quality of reconstruction also depends on the reconstruction software. In order to identify the performance of different software tools, we constructed 3D models using Agisoft® Photoscan®, Reality Capture[18] and COLMAP[19], [20], respectively. Fig. 17(a) presents the reconstructed 3D models using these three tools. These reconstructed models were registered (using Geomagic Design X) to the “reference” model, and their differences are presented in Fig. 17(b). In Fig. 18, the histograms of the absolute error regarding each vertex (in percentage) of these 3 registered models to the “reference” model are presented. The mean absolute error (MAE) of the model reconstructed by Agisoft® Photoscan® was found as 0.62 ± 0.28 mm. For the 3D models reconstructed by Reality Capture and COLMAP, the MAEs were 1.00 ± 1.48 mm and 0.82 ± 0.70 mm, respectively. Though in this case, the MAE of the model reconstructed by Agisoft® Photoscan® is slightly lower than the MAEs of models reconstructed by Reality Capture® and COLMAP®, in the context of product design, all reconstructed models can be used as the errors are much smaller than normal skin deformations [21].

Besides the software tool used for the reconstruction, the quality of images captured by the scanner is essential to the result. Sharp images with less noise and distortion offer a good basis for reconstructing a 3D model using photogrammetry. Many factors may influence the quality of the image, such as the environmental illumination, the lens, the CCD, the exposure time, and the focal length of the camera. Those factors were incorporated in the design of the scanner, e.g. the scanner was built as a soft box, the focal distance was optimized based on the parameters of the camera. However, there is still room for improvement, e.g. using better lenses, which can be considered as future research directions.

**Fig. 19:**
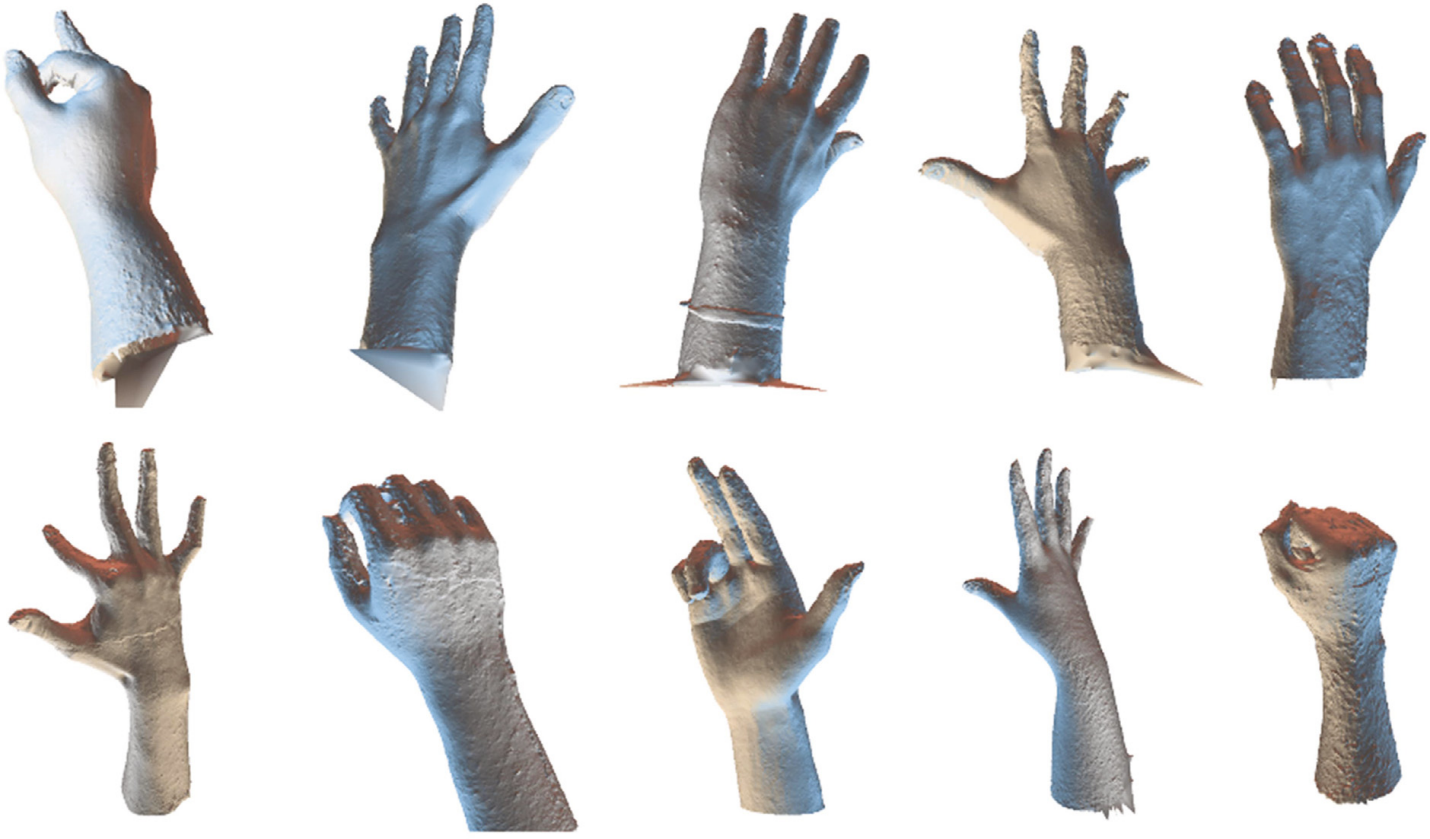
Some reconstructed hand models of different subjects in different postures.

Many volunteers were invited for scanning their hands and some examples are shown in Fig. 19. All hand models can be successfully reconstructed except some noise between fingers, which can be easily removed in the post-processing, e.g. using Geomagic Design X.

### Comparison with other scanners

Yunardi and Imandiri [22] designed a 3D scanner based on a line laser and cameras to capture the contour of the human wrist. Even though this scanner can be used to scan the human hand, the resolution of the result is low, and the average Root Mean Square Error (RMSE) between the reconstructed point and the reference point can reach up to 0.47 cm. Lee et al. [23] introduced a rotatory platform to scan the 3D body using multiple low-cost depth cameras. However, the operation principles, i.e. fixing cameras and rotating the scanning object, cannot be easily adapted to scan human hands, as it is difficult to keep the hand steady during the scanning process. Perhaps the 3D scanner presented by Straub and Kerlin [24] is the most similar system. It consists of fifty Raspberry Pi and cameras that are mounted on a large frame. However, this scanner was designed for scanning the full body, and the hands of the reconstructed model were not optimal.

**Fig. 20:**
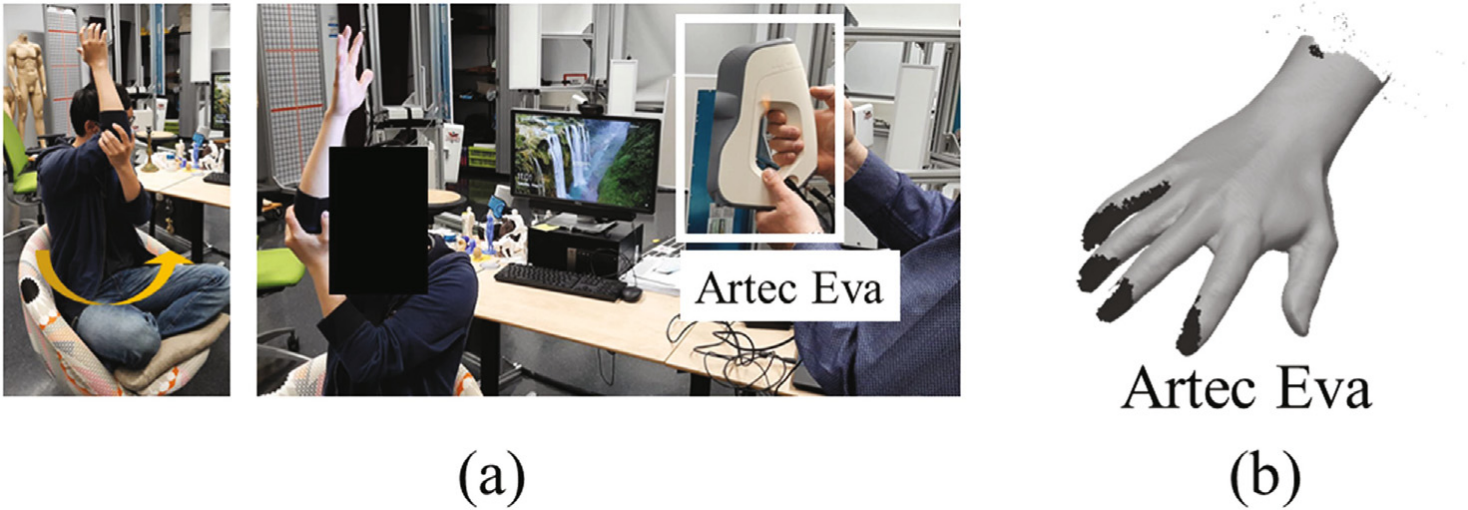
(a) The scanning process of using an Artec Eva® scanner. (b) The 3D scan captured by the Artec Eva® scanner.

To further verify the ability and usability of the designed hand scanner, we tried to acquire 3D scans of the human hand using an Artec Eva® scanner. Fig. 20(a) presents the scanning process where the Artec Eva® was held by the first researcher. A subject was invited to sit on a rotary chair and raise his hand over his head. Among the setups of rotating the scanner around the subject or rotating the chair for scanning the hand, we found that rotating the chair might be a better option. In the scanning process, while the subject was asked to keep the posture (incl. body and hand) as steady as possible, the second researcher (not in the picture) slowly rotated the chair based on the instructions of the first researcher. Using this method and after a few tries, it is possible to acquire the scan of the hand within 40~50 seconds. However, the quality of the scan strongly depends on the cooperation of the subject and two researchers. Fig. 20(b) presents one of the better scans among the results of a few tries. It can be observed that some parts around the tip of the fingers are still missing. As a comparison, the data acquisition time of the proposed scanner is very short (less than 80 ms), only one operator is needed, and the scanning results are always usable.

### Capabilities

The capabilities of the designed hand scanner are listed as following:

1. Modular design and can be easily and quickly built at an affordable price;
2. Fast image capturing of 50 cameras synchronized within 80 ms;
3. The mean absolute error of the reconstructed model reaches 0.62 ± 0.28 mm compared to the scans captured by the Artec Spider® scanner;
4. Wireless control of the process from a remote computer.

### Limitation

The design of the proposed hand scanner is modular, simple, and easy-to-build, it has been used successfully in personalized product design [25] and anthropometric studies [13]. However, there are still some limitations: 1) The scanner is designed and optimized for scanning the hand, even though other similar objects can also be scanned using this scanner, the 3D reconstruction result may not be as good as the hand; 2) The user-interface (UI) of the computer is simple, i.e. by typing commands in the terminal. It can be improved by a more user-friendly UI for a wider audience.

## Declaration of Competing Interest

The authors declare that they have no known competing financial interests or personal relationships that could have appeared to influence the work reported in this paper.
